# High early incidence of sepsis and its impact on organ dysfunction in burn trauma patients: a detailed and hypothesis generating study

**DOI:** 10.1093/burnst/tkae085

**Published:** 2025-02-10

**Authors:** Folke Sjoberg, David Greenhalgh, Moustafa Elmasry, Islam Abdelrahman, Ahmed T El-Serafi, Ingrid Steinvall

**Affiliations:** Department of Hand Surgery, Plastic Surgery, and Burns, Linköping University, Linköping 581 85, Sweden; Department of Biomedical and Clinical Sciences, Linköping University, Linköping 581 85, Sweden; Department of Burns, Shriners Children’s Northern California and Department of Surgery, University of California, Davis, Sacramento, CA 95817-2215, United States; Department of Hand Surgery, Plastic Surgery, and Burns, Linköping University, Linköping 581 85, Sweden; Department of Biomedical and Clinical Sciences, Linköping University, Linköping 581 85, Sweden; Department of Hand Surgery, Plastic Surgery, and Burns, Linköping University, Linköping 581 85, Sweden; Department of Biomedical and Clinical Sciences, Linköping University, Linköping 581 85, Sweden; Department of Biomedical and Clinical Sciences, Linköping University, Linköping 581 85, Sweden; Department of Hand Surgery, Plastic Surgery, and Burns, Linköping University, Linköping 581 85, Sweden; Department of Biomedical and Clinical Sciences, Linköping University, Linköping 581 85, Sweden

Burn injuries offer a valuable model for investigating the impact and therapeutic approaches for trauma in humans [1]. A distinct advantage in this context is the accurate determination of the injury’s size and timing, providing a robust basis for examining trauma-induced effects in humans.

A notable challenge following burn trauma is the emergence of infectious complications, potentially complicating the clinical scenario [2, 3]. Sepsis in burn patients poses a significant diagnostic challenge as the symptoms of severe burns parallels that of sepsis in the general population [3]. In cases of intermediate-sized burns with the patient mostly on a survival trajectory, the incidence of organ dysfunction typically peaks during the fluid loss period and the ensuing first week after the injury before gradually subsiding [4].

A pivotal aspect of early post-burn involves distinguishing the extent to which infectious complications influence the trauma response. Given that the trauma itself can induce organ dysfunction/failure, it becomes essential to discern whether any infectious complications are directly linked to organ failure or are merely a concurrent phenomenon. This distinction is crucial for differentiating between mere infection and sepsis, the latter being identified if there is a suspected infection inducing an organ failure level of 2 or higher, according to the Sequential Organ Failure Assessment (SOFA) and Sepsis-3 criteria [5].

This prospective single-center study aimed to evaluate the incidence of sepsis, as defined by the Sepsis-3 criteria [5], in the early stages (< 14 days post burn), and to compare this with organ dysfunction potentially attributable to the burn trauma itself in patients without an infection. A hypothesis, based on prior studies indicating high early sepsis rates [6], posits that the prevalence of sepsis, as per the Sepsis-3 criteria [5], may be higher than previously assumed and is the primary cause of early organ dysfunction. This higher rate also impacts the early trauma response as assessed by C-reactive protein (CRP). The occurrence of late sepsis was also examined, i.e. during the third week or later.

Patient included were consecutive in-hospital burn patients admitted to Linköping Burn Center, Sweden, from April 1, 2017, to October 31, 2019, with a population coverage of 5 million.

After admission, patients were resuscitated according to the Parkland formula. All patients received early (<5 days after injury) burn wound excision followed by temporary or permanent wound closure with autograft, allograft, or skin substitutes. No prophylactic antibiotics were given. Nutritional support was provided through early enteral nutrition, and ventilatory treatment by pressure-controlled settings as specified in the ARDSnet protocol [7]. The burn care procedures are protocolized and based on checklist methodology, which has repeatedly been previously reported [8].

The suspicion of infection was according to the discretion of the investigator. Relevant triggers were body temperature > 38.5, significantly increased plasma CRP and/or plasma-procalcitonin, observed wound infection, and/or positive Chest X-ray (CXR; infiltrates). The Sepsis-3 criteria for sepsis were used [5]. The diagnosis of sepsis and septic shock was divided into early sepsis, within 2 weeks or late sepsis during the third week or later. For the Acute Respiratory Distress Syndrome (ARDS), inhalation injury and Ventilator–associated pneumonia (VAP) diagnoses we applied the Berlin definition and VAP was defined as documented pneumonia based on the CXR findings, positive respiratory cultures. Inhalation injury was diagnosed by bronchoscopy.

During the period 292 patients were admitted, and 62 with a TBSA >10%, amongst those, 32 patients needed ICU care (Figure 1a, and Table 1). During the first three weeks, nine patients were free of sepsis (Non-Sepsis, **NS**), six patients were classified as having had sepsis only (Sepsis, **S**) and 17 had at least one episode of septic shock (Septic Shock, **SS**). The distribution of the burn wound sizes for the three groups are depicted in Figure S1 (see online supplementary file). Of the 24 patients with an early period of **S**, 14 also got a second period of **S**. Two patients only got a late septic episode, i.e. during the third week. No later septic shock episodes were registered (data not shown). Examining the overall SOFA score for each patient, there was a different trajectory between the three groups (**NS, S, and SS**; Figure 1e-i) (*p* < .001). Especially an early (Day 3) decline was observed for the **NS** group, (Figure 1e). Examining organ specific SOFA trajectories (Respiratory, Cardiovascular and Coagulation) significant differences were seen between the **NS** and the **S** group (Figure 1e-i). For the respiratory system normalization was noted within a week for the **NS** group whereas it for the **S** and **SS** patients remained impaired beyond 2 weeks post burn (*p* < .001).

**Figure 1:**
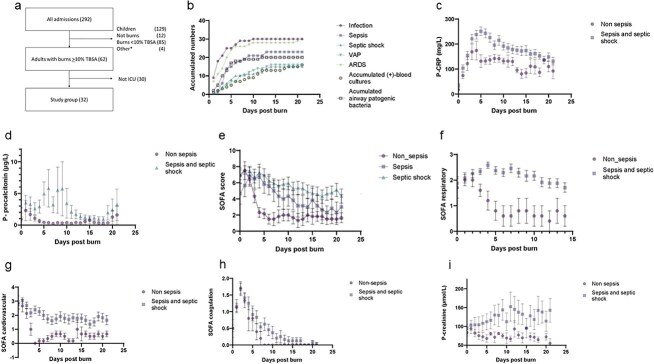
Study outline, infectious and sepsis effects. (**a**) Patient selection among in-hospital admissions during the period 1st April 2017—31st October 2019. *not included: Delayed admission, multitrauma, poor prognosis, renal failure. The cohort consisted of patients with a total body surface area (TBSA) burned of >10% who required ICU care (n = 32). Patient data was collected according to a preset standard prospective clinical treatment protocol, connected to a local registry at the center^4, 8, 10^. (**b**) Accumulated number of patients of the total cohort of 32 patients, that acquired infection, sepsis, septic shock, VAP, ARDS, (+)-blood cultures Table S2 (see online supplementary file); airway pathogenic bacteria Table S1 (see online supplementary file) and during the first 21 days after the burn injury. (**c**) Changes in CRP during the first 3 weeks for the non-septic patients and the sepsis/septic shock groups. Values as mean ± SEM. (*p* < .001, t-test/ANOVA). (**d**) Procalcitonin levels during the first 3 weeks after the burn for the non-septic patients and the sepsis/septic shock groups. Values as mean ± SEM.(*p* = .0125, t-test/ANOVA). (**e**) Overall SOFA scores for the first 3 weeks after the burn for the three groups, **NS**, **S** and **SS**. Values as mean ± SEM. (t-test/ANOVA, *p* <.001). (**f**) Respiratory SOFA score for the non-septic patients and the patients with sepsis/septic shock. (*p* < .001, t-test/ANOVA) values as mean ± SEM. (**g**) Cardiovascular SOFA score for the non-septic patients and the patients with sepsis/septic shock. (*p* < .001, t-test/ANOVA) values as mean ± SEM. (**h**) Coagulation SOFA score for the non-septic patients and the patients with sepsis/septic shock. (*p* < .001, t-test/ANOVA) values as mean ± SEM. (**i**) Creatinine for the non-septic patients and the patients with sepsis/septic shock. (*p* < .001, t-test/ANOVA) values as mean ± SEM. *ARDS* Acute Respiratory Distress Syndrome, *CRP* C-reactive protein, *SOFA* Sequential Organ Failure Assessment, *ICU* intensive care unit, *NS* Non-Sepsis, *SS* Septic Shock, *S* Sepsis

**Table 1:** Details of the patients with a TBSA % > 10% by treatment group

	**Study group (ICU care)**	**Not ICU**	***p***
Patients	32	30	
Sex, male, *n* (%)	23 (72)	23 (77)	0.67
Age, years	49.0 (34.0–69.5)	55.0 (34.0–71.0)	0.85
Hospital stay, days	46.5 (34.0–71.0)	23.5 (17.0–32.0)	<0.001
Burn size, %TBSA	34.8 (26.3–40.5)	12.5 (11.5–17.0)	<0.001
Superficial dermal burn, BSA%	0.5 (0.0–5.4)	4.2 (1.0–10.0)	0.03
Deep dermal burn, BSA%	14.3 (2.8–27.3)	6.3 (1.0–9.5)	0.01
Full-thickness burn, BSA%	8.0 (0.0–20.5)	0.0 (0.0–2.0)	0.003
Mortality, *n* (%)	4 (12)	1 (3)	0.36

Data are presented as median (25th–75th centile) or n (%). Mann–Whitney *U* and chi squared or Fisher’s exact tests was used as appropriate. *TBSA%* percentage total body surface area, *ICU* intensive care unit, *BSA* body surface area

The study unveils several innovative and noteworthy discoveries. Primarily, within an ICU Burn cohort devoid of prophylactic antibiotics, a markedly elevated initial infection rate of 91% was observed. This correlates with sepsis and septic shock at rates of 50% and 31%, respectively, during the initial week. Secondly, comparing patients not affected by sepsis and those experiencing sepsis or septic shock revealed a distinct divergence. In the non-septic group, the overall organ failure subsided within a week. Conversely, in the sepsis and septic shock group, this extended throughout the majority of the initial three weeks. This prolonged decline was accompanied by cumulative rates of ARDS and VAP reaching 88% and 47% within a fortnight, respectively. Thirdly, an intriguing observation is the normalisation of organ failure scores post one week in the non-septic group. This suggests that the trauma-induced inflammatory response, e.g. depicted by CRP, a potential catalyst for early organ impairment subsides before the end of the first week. Subsequent organ dysfunction appears predominantly driven by sepsis or septic shock. The observed mortality rate, which was comparatively low, 12%, aligned with the anticipated outcomes based on age and TBSA%. It also needs to be emphasized that sepsis occurred late as well, then most commonly (n = 14) in the patients that already had had a bout of sepsis. Only two patients remained free of sepsis, and organ failure throughout the care trajectory. Another interesting observation was the close correlation between positive blood cultures and septic shock. The data presented suggests that infection already at the end of the first week is a crucial factor for organ failure, whereas organ failure in the first 4 days, such as often seen with e.g. ARDS, can largely be attributed to trauma-related effects. A vital question is whether early antibiotic treatment could have reduced the frequency of infections and organ dysfunction.

There is now an increasing consensus that the Sepsis-3 criteria [5] are the most suitable also for the burn setting [9]. In this study, we relied on these criteria, identifying a significant impact on organ failure (> 2 SOFA points) occurring within 2–3 days of an infection. This was typically the case; however, there were instances where, in the absence of infection, the SOFA score would have decreased after Days 3–4, and this decrease did not occur, resulting in no clear increase but rather in a failure to decrease. In these cases, coinciding with an infection, we may have missed a sepsis diagnosis, thus posing a risk of underreporting sepsis. For the diagnosis of septic shock, we based it on the presence of sepsis followed by the need for inotropic support and a lactate level above 2 mmol/L. Here, the risk lies in not conducting frequent enough lactate measurements, leading to a potentially falsely low registration of septic shock.

The markers for infection, sepsis, and septic shock that have been deemed significant include elevated body temperature, P-CRP, P-Procalcitonin, and S-Lactate. With the diagnostic criteria used for infection, sepsis, and septic shock in this study, the outcomes of these markers were clear and closely aligned with expectations.

Regarding organ failure in burn injuries, there is a notable paucity of recent, modern studies specifically addressing the prevalence of early organ failure following the injury. Recently, early organ failure, particularly due to early respiratory failure and its causes (i.e. inhalation injuries and subsequent development of ARDS, secondary either to burn trauma or infection), has however garnered significant interest [10]. The results of this study predominantly suggest that the earliest respiratory failure may be attributable to trauma-induced inflammation in the first week. However, if it persists beyond this period, it is more likely to be maintained by infection and sepsis. This finding is novel in this context.

The primary limitation of this study is the number of patients, particularly when conducting subgroup analyses. Furthermore, although the average extent of injury was substantial, a mean TBSA% at 34.8%, with full-thickness burns constituting 22.3%, the cohort contained few injuries exceeding 60%. Despite this, clear differences were observed in almost all outcome parameters examined, supporting the conclusions drawn from the study.

Data from this study indicates that the frequency of infection and sepsis soon after a burn injury is higher than previously documented and that the organ failure occurring after the first week post-injury mainly is dependent on sepsis, whereas early respiratory failure (ARDS) in the beginning of the first week, is a consequence of the burn trauma.

## Supplementary Material

Figure_S1_tkae085

Table_S1_and_S2_tkae085
