# Supplementary figures and images for: High early incidence of sepsis and its impact on organ dysfunction in burn trauma patients: a detailed and hypothesis generating study
Source: Burns Trauma. 2025 Feb 10;13:tkae085. doi: 10.1093/burnst/tkae085 (PMC11808796; doi:10.1093/burnst/tkae085)

a)

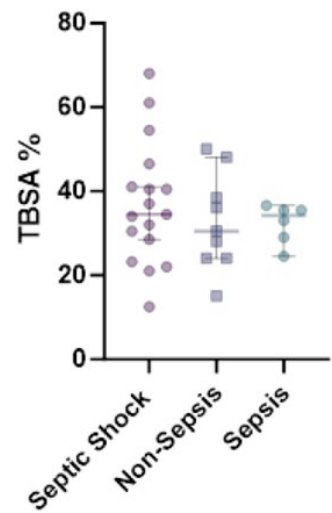

b)

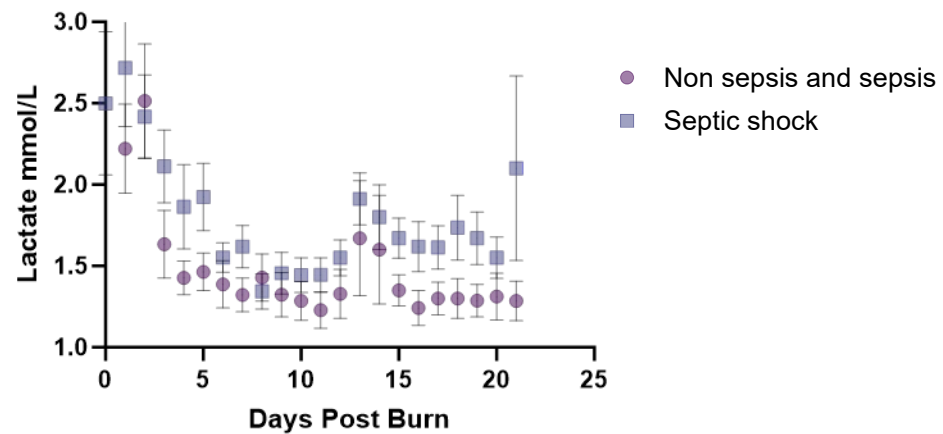

c)

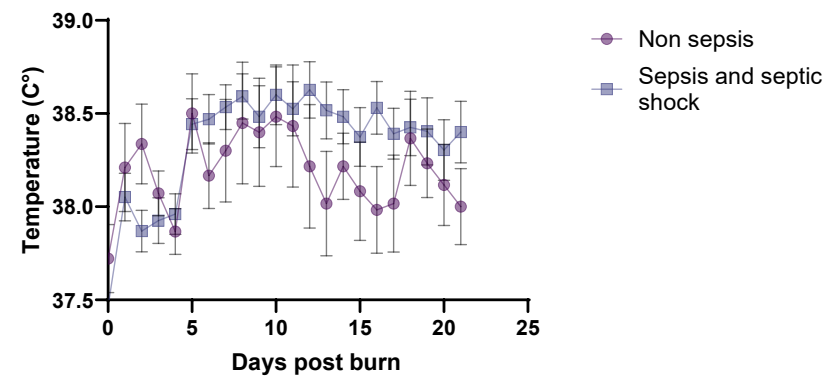

Supplement: Figure_S1_tkae085 [file figure_s1_tkae085.pdf]
