# Supplementary material for: High early incidence of sepsis and its impact on organ dysfunction in burn trauma patients: a detailed and hypothesis generating study
Source: Burns Trauma. 2025 Feb 10;13:tkae085. doi: 10.1093/burnst/tkae085 (PMC11808796; doi:10.1093/burnst/tkae085)
Supplement: Table_S1_and_S2_tkae085 [file table_s1_and_s2_tkae085.docx]

## Supplemental Material

## Cultures

The cultures taken from the respiratory tract as well as in the blood positive culture were most often Staphyloccocci and Streptococci. No Pseudomonas were registerered in these compartments. **(Table S1 and S2)**

| **Table S1 – Pulmonary brush cultures** | |
| --- | --- |
| *Staphylococcus aureus* | 7 |
| *Streptococcus pneumoniae* | 5 |
| *Haemophilus influenzae* | 4 |
| *Alfa-streptococci* (*S. mitis* group*)* | 2 |
| *Serratia marcescens* | 2 |
| *Enterobacter cloacae* | 1 |
| *Staphylococcus epidermidis* | 1 |
| *Klebsiella pneumoniae* | 1 |
| *Proteus mirabilis* | 1 |
| *Escherichia coli* | 1 |
| *Acinetobacter baumani* | 1 |
| Number of positive cultures | |

| **Table S2 – Blood cultures** | | |
| --- | --- | --- |
| *Staphylococcus epidermidis* | 6 |  |
| *Staphylococcus aureus* | 4 |  |
| Enterococci | 3 |  |
| *Serratia marcescens* | 2 |  |
| *Acinetobacter baumani* | 1 |  |
| *Corynebacterium striatum* | 1 |  |
| *Klebsiella variicola* | 1 |  |
| *Alfa-streptococci* (*S. mitis* group) | 1 |  |
| *Propionibacterium acnes* | 1 |  |
| Number of positive cultures | | |
